# Effect of Mixed Cultures of Yeast and Lactobacilli on the Quality of Wheat Sourdough Bread

**DOI:** 10.3389/fmicb.2019.02113

**Published:** 2019-09-10

**Authors:** Dan Xu, Yao Zhang, Kaixing Tang, Ying Hu, Xueming Xu, Michael G. Gänzle

**Affiliations:** ^1^National Engineering Laboratory for Cereal Fermentation Technology, State Key Laboratory of Food Science and Technology, School of Food Science and Technology, Jiangnan University, Wuxi, China; ^2^Department of Agricultural, Food and Nutritional Science, University of Alberta, Edmonton, AB, Canada; ^3^College of Bioengineering and Food Science, Hubei University of Technology, Wuhan, China

**Keywords:** sourdough bread, mixed culture, CO_2_ capacity, bread volume, glutenin macropolymer, volatiles

## Abstract

In this study, mixed starter cultures of yeast and lactobacilli were used for type I sourdough bread making to evaluate their ability to improve bread quality and increase the amount of flavor volatiles. *Kazachstania humilis*, *Saccharomyces cerevisiae*, *Wickerhamomyces anomalus*, and *Lactobacillus sanfranciscensis* DSM20451^T^ and *Lactobacillus sakei* LS8 were used in different combinations to ferment wheat sourdough. *S. cerevisiae* produced the highest amount of CO_2_ among all strains and thus enhanced bread volume and crumb texture. *S. cerevisiae* also increased the free thiol level in bread dough, and this study confirms that thiol accumulation was not strongly related to the content of the glutenin macropolymer (GMP) or bread volume. The role of thiol exchange reactions on bread quality differs between long fermentation sourdough and straight dough with baker’s yeast only. The influence of different starter cultures on wheat sourdough bread volatiles was established by using head space solid-phase microextraction and gas chromatography/mass spectrometry analysis (SPME-GC/MS). The sourdough breads fermented with a combination of lactobacilli and yeast had a more complex profile of volatiles, particularly with respect to esters.

## Introduction

Sourdough fermented with lactic acid bacteria (LAB) and yeasts has traditionally been used as a leavening agent in bread making; in recent years, the use of sourdough or sourdough products as baking improver has re-emerged as standard method in bread production (Gänzle and Zheng, 2018). In industrial baking, sourdough is generally used in combination with baker’s yeast, *Saccharomyces cerevisiae*, to achieve dough leavening. Application of sourdough in wheat baking improves the flavor, texture, and enhances the storage-life by delayed staling and fungal spoilage (Hansen and Schieberle, 2005; Gänzle, 2014; Gobbetti et al., 2018). These benefits are achieved without additives and depend on enzymatic conversion and metabolites of LAB and yeasts during fermentation (Hansen and Schieberle, 2005). Traditional sourdoughs (Type I sourdoughs) harbor *Lactobacillus sanfranciscensis* in association with *Kazachstania* species, predominantly *Kazachstania humilis*, while industrial sourdoughs (Type II sourdoughs) are populated by vertebrate host associated *Lactobacillus* sp. of the *L. reuteri* group and *L. delbrueckii* group, with or without yeasts present (De Vuyst et al., 2016; Gänzle and Zheng, 2018). The composition of the flour and the metabolic interactions between LAB and yeasts greatly influences the sourdough leavening, because yeast strains produce different amounts of carbon dioxide (Gobbetti et al., 1995; Häggman and Salovaara, 2008). Bread crumb flavor is influenced by odorants present in the flour and by aroma compounds produced in microbial or enzymatic reactions at the dough stage (Czerny and Schieberle, 2002; Hansen and Schieberle, 2005). Sourdough lactobacilli influence the bread flavor mainly through conversion of carbohydrates and amino acids to flavor compounds or flavor precursor compounds (Gänzle, 2014). In comparison to LAB sourdough have a greater capacity to produce flavor volatiles, and different yeast strains differ with regards to the production of flavor volatiles (Damiani et al., 1996; Liu et al., 2018).

Gluten polymerization and proteolysis during sourdough fermentation are key determinants for bread quality. Protein hydrolysis releases amino acids as taste compounds or flavor precursors but also degrades the glutenin macropolymer (GMP). The amino acids such as glutamate improve the taste of sourdough bread; other amino acids including ornithine, leucine and phenylalanine are flavor precursors, which are converted into 2-acetyl-pyrroline, 3-methyl-butanol, and 2-phenylethanol, respectively, (Hansen and Schieberle, 2005; Gänzle, 2014). The GMP in wheat doughs mediates dough hydration and gas retention which highly correlates to bread volume (Wieser and Kieffer, 2001; Goesaert et al., 2005; Wieser, 2007). At kneading, inter- and intra-chain disulfide exchange reactions polymerize glutenin subunits (Singh and Macritchie, 2001). During dough rest and proofing, gluten properties are modified through thiol-exchange reactions (Weegels et al., 1996). Thiol-disulfide exchange reactions during bread making are impacted by microbial accumulation of glutathione (Jänsch et al., 2007; Tang et al., 2017), and by production of CO_2_, and organic acids that also impact gluten functionality (Clarke et al., 2004; Rezaei et al., 2015). In turn, viscoelastic properties of wheat dough as determined by gluten proteins are critical for the expansion of gas bubbles and the retention of CO_2_ (Verheyen et al., 2015).

The accumulation of glutathione is mediated by glutathione reductase activity of *L. sanfranciscensis*, which reduces oxidized glutathione at the expense of NAD(P)H that is generated in primary carbon metabolism (Jänsch et al., 2007). The role of LAB on reduction or oxidation of thiols in wheat doughs has been thoroughly investigated with *L. sanfranciscensis*, *L. reuteri*, and *L. sakei* as model organisms (Jänsch et al., 2007; Tang et al., 2017) but few data are available on the role of yeasts. Dried baker’s yeast significantly contributed to the level of reduced thiols in bread dough (Xu et al., 2018). *S. cerevisiae* accumulates glutathione in response to oxidative stress during desiccation (Musatti et al., 2013). It is unclear, however, whether active cells of *S. cerevisiae* have the same contribution to thiol levels in bread dough, and whether other yeast species that are more typically observed in sourdough exhibit a comparable accumulation of thiols. This study therefore aimed to compare different yeast species isolated from sourdough with respect to their contribution to CO_2_ production and thiol accumulation. Sourdough yeasts were propagated together with *L. sanfranciscensis* or *L. sakei* in a model type I fermentation (Tang et al., 2017). In addition, the influence of baker’s yeast and sourdough yeasts to the profile of bread volatiles was investigated.

## Materials and Methods

### Strains, Media, and Growth Conditions

The lactobacilli and yeast strains used in the study were *L. sanfranciscensis* DSM20451^T^, *Lactobacillus sakei* LS8, *K. humilis* FUA4001, *S. cerevisiae* FUA4018, and *W. anomalus* FUA4025. All three yeast strains were isolated from traditional sourdoughs that are used for dough leavening in Canada (unpublished) or Italy (Ripari et al., 2016). Lactobacilli were cultivated anaerobically in modified De Man, Rogosa and Sharp medium (mMRS) at 32°C (Tang et al., 2017). Yeasts were cultivated aerobically (180 rpm and 30°C) in Yeast Peptone Dextrose medium (YPD, containing 1% yeast extract, 2% peptone, 2% dextrose, 0.01 chloramphenicol); 1.5% of agar was added for solid medium (Xu et al., 2019). Lactobacilli were subcultured twice with 1% inoculum in mMRS broth for 16–20 h; yeasts were subcultured with 4% inoculum in YPD broth for 20–24 h. Inocula for sourdough fermentation were prepared by washing of overnight cultures of yeasts or lactobacilli in sterile tap water, followed by re-suspension in sterile tap water to achieve a cell count of around 10^6^ and 10^7^ CFU/mL for yeasts and lactobacilli, respectively. The combinations of LAB and yeasts used are reported in Table 1.

**TABLE 1:** pH of sourdough, bread dough and bread.

**Codes of breads**	**Combined starters**	**pH**					
		**Stage I**	**Stage II**	**Mix**	**Rest**	**Proof**	**Bread**
K1 (+)^a)^	*K. humilis*	3.910.07^abc^	4.230.10^ab^	5.000.05^cde^	4.710.09^ef^	4.680.05^de^	4.690.05^de^
K1 (*−*)	*L. sanfranciscensis*			5.070.03^bc^	4.800.08^cde^	4.700.08^de^	4.730.04^de^
K2 (+)	*K. humilis*	3.760.11^cdef^	4.050.08^cd^	4.890.06^ef^	4.680.08^f^	4.610.09^ef^	4.610.07^e^
K2 (*−*)	*L. sakei*			4.930.08^def^	4.730.07^cdefg^	4.710.05^de^	4.710.04^de^
S1 (+)	*S. cerevisiae*	4.010.03^a^	4.390.06^a^	5.160.07^b^	4.840.05^c^	4.770.02^cd^	4.870.11^bc^
S1 (*−*)	*L. sanfranciscensis*			5.150.10^b^	5.010.05^b^	4.910.04^b^	4.990.08^ab^
S2 (+)	*S. cerevisiae*	3.720.03^def^	4.120.03^bc^	5.050.03^bcd^	4.710.04^ef^	4.550.07^f^	4.600.14^e^
S2 (*−*)	*L. sakei*			5.050.06^bcd^	4.790.09^cdef^	4.760.05^cd^	4.810.09^cd^
W1 (+)	*W. anomalus*	3.920.09^ab^	4.120.03^bc^	4.980.06^cdef^	4.810.04^cde^	4.730.09^cde^	4.650.08^e^
W1 (*−*)	*L. sanfranciscensis*			5.050.05^bcd^	4.980.09^b^	4.850.08^bc^	4.800.09^cd^
W2 (+)	*W. anomalus*	3.830.13^bcd^	4.010.07^cd^	4.930.06^def^	4.720.06^def^	4.620.07^ef^	4.690.03^de^
W2 (*−*)	*L. sakei*			4.970.05^cdef^	4.810.05^cde^	4.780.05^cd^	4.820.05^cd^
L. sf (+)	*L. sanfranciscensis*	3.620.06^f^	3.850.08^e^	4.750.05^g^	4.630.02^g^	4.340.10^g^	4.370.06^f^
L. sk (+)	*L. sakei*	3.660.04^ef^	3.880.06^e^	4.740.14^g^	4.780.04^cdef^	4.510.04^f^	4.590.05^e^
CA (+)	CA control^b)^	3.800.10^bcde^	3.930.06^de^	4.860.05^fg^	4.830.03^cd^	4.610.09^ef^	4.630.07^e^
SD (+)	Straight dough	n.a.	n.a.	5.530.04^a^	5.420.03^a^	5.340.02^a^	5.480.04^a^

*Data are presented as means ± standard deviation of triplicate independent fermentation and baking trials. Values in the same column differ significantly if they do not share a common superscript (*P* < 0.05) (*n* = 3). ^*a*^ (+) indicates addition of baker’s yeast to the bread dough; (−) no addition of baker’s yeast; ^*b*^ CA, chemically acidified; and n.a., not available.*

### Sourdough Fermentation and Bread Making

This type I sourdough bread making process was performed according to Tang et al. (2017) with slight modifications. Commercial wheat flour (10 g) was mixed with 10 mL of inoculum and fermented at 30°C for 16 h (Stage I). The second fermentation stage was started with stage I sourdough and addition of 20 g of wheat flour and 20 g of tap water, followed by fermentation at 30°C for 2.5 h. Bread dough was prepared with 70 g of wheat flour, 30 g of tap water, 2 g of salt, 2 g of sugar, 0.5 g of dried commercial baker’s yeast, and 60 g of stage II sourdough. Doughs were mixed for 6 min, followed by resting for 1 h at 30°C, shaping and proofing for 1 h at 30°C. Bread was baked at 180°C for 25 min. Control breads were prepared as follows: Straight dough bread without sourdough (SD+), bread made with chemically acidified sourdough (CA+), and mixed starter fermented sourdough bread without addition of baker’s yeast (K1−, K2−, S1−, S2−, W1−, and W2−). The chemically acidified sourdough was acidified to a pH value of 3.80 ± 0.10 with 0.27% (v/w) of a mixture of acetic acids (100% w/w) and lactic acid (85% w/w) in a ratio of 1:4 (v/v), and incubated under the same conditions as stage I. For stage II, 20 g of wheat flour and 20 mL of tap water were added together with 0.12% of the mixed acids to a pH value of 3.99 ± 0.10. Straight dough bread was prepared by mixing of 100 g of wheat flour, 60 g of tap water, 2 g of salt, 2 g of sugar, 0.5 g of baker’s yeast. The bread dough without addition of baker’s yeast was prepared by mixing of 70 g of wheat flour, 30 g of tap water, 2 g of salt, 2 g of sugar, and 60 g of stage II mixed starter fermented sourdough. Resting, proofing, and baking of the dough was carried out as described as above.

### Determination of pH and Cell Counts CFU/g During Fermentation

Fresh dough samples were used for pH determination; 1 g of sample was mixed with 9 g of 18 MΩ water and the pH was measured with a glass electrode after vortexing for 1 min. For cell counts, fresh sourdough samples were diluted to three appropriate concentrations, then determined by plating them on mMRS or YPD agar. The plates were incubated anaerobically/aerobically at 32°C/30°C for 2–3 days. To ensure the inoculated strains dominated the dough microbiota, a uniform colony morphology that matched the corresponding strain was observed in all sourdough samples throughout stage I and stage II (Lin and Gänzle, 2014; Zhao et al., 2015).

### Measurement of CO_2_ Production

A rheofermentometer F3 (Chopin, Villeneuve-La-Garenne Cedex, France) measured the CO_2_ release of the dough. The amount of ingredients was scaled up to make a 300 g bread dough after kneading. The dough was placed immediately in the fermentation jars with a 2000 g cylindrical weight on the dough. The test was conducted at 30°C for 2 h. Each group was carried out in triplicate independent experiments.

### Determination of Bread Specific Volume and Crumb Hardness

After baking, breads were cooled at room temperature for 1 h, weighed, and the loaf volume was measured by the rapeseed displacement method (AACC Method 10-05.01). The specific volume was calculated by dividing the volume to weight and expressed as mL/g.

Bread crumb hardness was determined using a TA.XTPlus model texture analyzer (Stable Micro System Co., Ltd., Surrey, England). One hour after baking, the bread was cut into slices of 10 mm thickness, and determined with a modified AACC method 74-09 (AACC International, 2000). The parameters for testing were set as following: the probe type was P/25; the pre-test speed was 3.00 mm/s; testing speed was 1.00 mm/s; post-test speed was 5.00 mm/s; compressed twice with 40% compression, and the interval time was 10 s.

### Determination of Free Thiol Groups and Total Peroxides

Ellman’s reagent was used as described to quantify the free thiol groups in dough samples (Jänsch et al., 2007). Quantification of free thiols includes low molecular weight compounds other than glutathione (Jänsch et al., 2007; Tang et al., 2017) and is therefore a more reliable indicator for overall thiol-exchange reactions in dough. Freeze-dried sample (40 mg) was mixed with 4 mL sodium dodecyl sulfate (SDS)-TGE buffer, then extracted by shaking at room temperature for 30 min. After adding 0.04 mL DTNB-TGE buffer, the samples were incubated for another 30 min in the dark. After centrifuging, the absorbance of supernatant was measured at 412 nm. Reduced glutathione was used to establish a standard curve.

The protocol to determine the total peroxides was based on Liao et al. (2007). Fresh dough samples (1 g) were vortexed with 2 mL deionized water for 3 min with glass beads. After centrifuging with 5000 × *g* at 4°C for 5 min, the supernatant was mixed with equal volume of potassium dichromate (5%) in acetic acid solution (1:3, v/v), then heated at 80°C for 10 min. The absorbance of the cooled down samples were measured at 570 nm. H_2_O_2_ was used to establish a standard curve.

### Quantification of SDS and SDS-Dithiothreitol (DTT) Soluble Proteins by Size Exclusion Chromatography

The influence of dough microbiota on the polymerization of gluten proteins was assessed by sequential extraction of proteins from sourdoughs and bread dough with SDS and SDS-DTT, followed by proteins quantification with SEC (Thiele et al., 2003; Xu et al., 2018). SEC was conducted on a LC system (Shimadzu, Kyoto, Japan) with a Shodex Protein KW-804 column (Showa, Kyoto, Japan) and a UV detector set at 214 nm. The SDS soluble proteins and SDS-DTT soluble proteins were extracted from sourdough and dough as described (Thiele et al., 2003). Lyophilized samples (0.1 g) were extracted with 1 mL of 50 mM sodium phosphate buffer (pH 6.9) containing 2% SDS at room temperature for 1 h. The supernatant containing SDS soluble proteins was collected after centrifuging. The SDS-DTT soluble proteins corresponding to the GMP in the insoluble pellet were extracted with the same buffer in the presence of 1% DTT at room temperature for another 1 h. The samples were filtered (0.22 μm) before injection on the SEC column and elution with 0.2% SDS in 50 mM sodium phosphate (pH 6.9) at room temperature at a flow rate of 0.7 mL/min. Peak areas were normalized to the corresponding peak area of the chemically acidified dough of the same batch after stage I (Xu et al., 2018).

### Relative Quantitation of Bread Volatiles

Volatile compounds were extracted by head-space solid phase microextraction (HS-SPME) and detected with gas chromatography-mass spectrometry (GC-MS). To extract volatiles, 2 g of bread crumb sample was put into a 20 mL headspace vial with a teflon-lined septum fit at its top. The SPME needle was pierced into the vial, which was then immersed in a 60°C water bath. The SPME fiber in the needle (2 cm–50/30 mm DVD/Carboxen/PDMS Stable Flex Supelco, Bellefonte, PA, United States) was then exposed to the headspace for 30 min. After extracting, the SPME fiber was inserted into the injector port for thermal desorption for 7 min.

The GC analyses were performed using a Shimadzu model SPL-2010 PLUS gas chromatograph equipped with an Agilent DB-WAX122-7032 (30 m × 0.25 mm × 0.25 μm) column coupled to a Shimadzu GCMS-QP2010 Ultra mass spectrometer. The GC temperature program was set as following: 40°C held for 3.5 min, then increased by 5°C/min to 90°C and held again for 5 min, then increased by 12°C/min to 220°C, where it was held again for 7 min. The flow rate of helium was 0.8 mL/min. For the MS program, the injector temperature was 280°C. The mass spectra were recorded by electronic impact (EI) at 70 eV, and the electron source temperature was 200°C. The mode was used to scan all the compounds in the range 33–495 m/z. Volatile compounds were identified by comparing with retention indices (RI) calculated with a standard mixture of paraffin homologous C10 to C25 (J&K Chemical Co., Ltd., Shanghai, China) and MS data obtained from MS libraries (NIST/WILEY/REPLIB/MAINLIB, 2005), or published elsewhere. The relative concentration of volatile compound was represented with its peak area.

### Statistical Analysis of Data

Sourdough fermentation and bread making were carried out in triplicate independent experiments. SE-HPLC analyses were performed with duplicate samples while all the other analyses were performed with triplicate samples. Significant differences (*P* < 0.05) and variance were calculated by one way analysis of variance (ANOVA) using SPSS software (version 20 for Windows, SPSS Inc., Chicago, IL, United States), and error bars indicate standard deviation. In addition, a principle component analysis (PCA) using SIMCA-P 11.5 software (Umetrics, Umea, Sweden) was carried out on the area of the volatile compounds extracted from different extracts, to characterize samples differences on aroma release.

## Results

### Characterization of Sourdough and Sourdough Bread

The pH and cell counts were determined to monitor fermentation microbiota of sourdoughs (Tables 1, 2). The initial pH value of the wheat flour was 5.65 ± 0.04, and decreased to 3.72–4.39 after stage I and stage II, respectively. The pH of sourdoughs fermented with lactobacilli was lower when compared to sourdough fermented with the same strain in association with yeasts; this difference was particularly significant for sourdoughs fermented with *L. sanfranciscensis*. The pH value of chemically acidified dough remained at 3.80–3.93. After mixing, the pH value of bread doughs prepared with sourdough was around 5.0, about half a pH unit lower than that of straight dough (5.5). Microbial activity during dough rest and proofing further reduced the pH of sourdough breads to values between 4.4 and 5.0; the pH of bread produced with the straight dough was 5.5. The microbial cell counts of chemically acidified doughs were below 3 Log CFU/g after all fermentation stages. The cell counts of yeasts and lactobacilli in stage I or stage II sourdoughs ranged from 6.70 to 7.98 Log CFU/g and 8.12 to 9.37 Log CFU/g, respectively. Association with *L. sakei* or *L. sanfranciscensis* did not impact the cell counts of yeasts; cell counts of *L. sanfranciscensis* grown alone were about 1 Log CFU/g higher when compared to sourdoughs where the same strain was inoculated in association with yeasts.

**TABLE 2:** Cell count during sourdough fermentation stages.

**Bread code**	**Combined starters**	**Viable cell count (log CFU/g)**			
		**Stage I (LAB)**	**Stage I (yeast)**	**Stage II (LAB)**	**Stage II (yeast)**
K1	*K. humilis* *L. sanfranciscensis*	8.410.06^d^	7.080.17^b^	8.150.14^c^	6.700.31^c^
K2	*K. humilis* *L. sakei*	8.850.31^bc^	7.130.18^b^	8.600.09^b^	6.720.23^c^
S1	*S. cerevisiae* *L. sanfranciscensis*	8.250.17^d^	7.090.15^b^	8.120.32^c^	6.700.35^c^
S2	*S. cerevisiae* *L. sakei*	9.010.14^abc^	7.650.24^a^	8.610.13^b^	7.020.13^bc^
W1	*W. anomalus* *L. sanfranciscensis*	8.620.25^cd^	7.710.22^a^	8.570.22^b^	7.360.11^ab^
W2	*W. anomalus* *L. sakei*	9.210.40^ab^	7.980.11^a^	8.790.11^ab^	7.700.27^a^
L. sf	*L. sanfranciscensis*	9.370.27^a^	n.a.	9.160.35^a^	n.a.
L. sk	*L. sakei*	9.270.11^ab^	n.a.	8.940.15^ab^	n.a.
CA	CA control^a)^	<3^e^	<3^c^	<3^d^	<3^d^
SD	Straight dough	n.a.	n.a.	n.a.	n.a.

*Data are presented as means ± standard deviation of triplicate independent fermentation and baking trials. Values in the same column differ significantly if they do not share a common superscript (*P* < 0.05) (*n* = 3). ^*a*^ CA, chemically acidified; n.a., not available.*

### Influence of Dough Microbiota on CO_2_ Formation and Bread Specific Volume and Crumb Hardness

**FIGURE 1:**
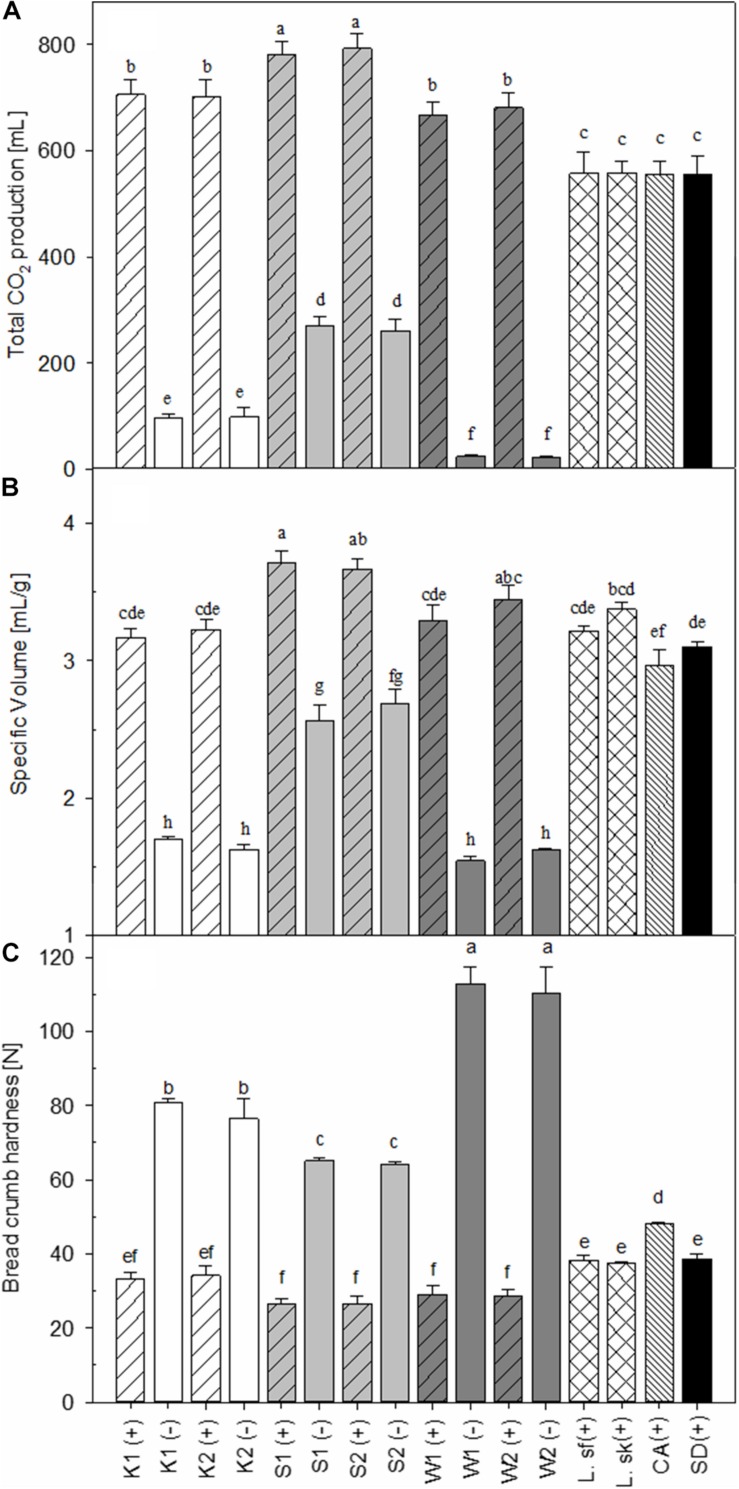
Total CO_2_ production of bread dough **(A)**, specific volume of bread **(B)**, and bread crumb hardness **(C)**. Bars that do not share a common lowercase letter differ significantly (*P* < 0.05) (*n* = 3). The inocula used for sourdough fermentation were abbreviated as follows: K, *K. humilis*, S, *S. cerevisiae*, W, *W. anomalus*, L. sf, *L. sanfranciscensis*, L. sk, *L. sakei*, CA, chemically acidified control, SD, straight dough control, (+) and (–) indicate addition of baker’s yeast at the bread dough stage, or not; 1 and 2 indicate combination with *L. sanfranciscensis* and *L. sakei*, respectively.

The leavening power of the different sourdough yeasts was assessed by determination of CO_2_ production in bread dough (Figure 1A), bread volume (Figure 1B) and crumb hardness (Figure 1C). The gas production was the highest in bread doughs prepared with sourdough and baker’s yeast (Figure 1A), particularly in sourdoughs fermented with *S. cerevisiae*. Intermediate gas production was observed in bread dough fermented with baker’s yeast alone or in combination with lactobacilli. CO_2_ production in bread dough without addition of baker’s yeast decreased on the order *S. cerevisiae* > *K. humilis* > *W. anomalus*. Fermentation with the heterofermentative *L. sanfranciscensis* or the homofermentative *L. sakei* did not influence CO_2_ production in bread dough, indicating that CO_2_ production was mainly attributable to the combination of yeast and LAB.

The specific volume of bread prepared with yeast-containing sourdoughs corresponded to the CO_2_ volume produced during fermentation (Figure 1B), while the crumb hardness was inversely related to CO_2_ production and bread volume (Figure 1C). The highest specific volume was obtained after the addition of *S. cerevisiae* and *L. sanfranciscensis* fermented sourdough (S1+); low bread volumes and a corresponding high crumb hardness was observed in sourdough breads produced without baker’s yeast. Among those breads produced without baker’s yeast, sourdoughs containing of *S. cerevisiae* and *L. sanfranciscensis* generated the highest volume and the lowest bread hardness (Figures 1B,C). Remarkably, the hardness of bread prepared with chemically acidified dough was higher when compared to other breads produced with unyeasted sourdough (*L. sanfranciscensis* or *L. sakei*) or bread produced with a straight dough process although the gas production during resting and proofing was similar (Figures 1B,C).

### Influence of Dough Microbiota on Thiol Groups and Total Peroxides in Dough

The free thiol content indicates breakdown and formation of disulfide bonds and thus links to the polymerization of gluten proteins. Peroxides that are generated in dough by enzymatic and microbial conversions promote the formation of disulfide bonds and reduce the content of free thiols (Joye et al., 2009). The free thiols (SH) and peroxides were measured after each step during the type I sourdough bread making process. The thiol content was the lowest in chemically acidified sourdoughs and the highest in sourdoughs fermented with *S. cerevisiae* (Table 3). The thiol content of bread dough with addition of different sourdoughs was generally not significantly different; only bread dough produced with sourdoughs fermented with *S. cerevisiae* and *L. sanfranciscensis* exhibited a higher thiol content when compared to chemically acidified and straight dough controls (Table 3).

**TABLE 3:** Concentration of SDS extracted free thiol in sourdoughs and bread dough.

**Bread code**	**Combined starters**	**Free thiol content (μmol/g)**				
		**Stage I**	**Stage II**	**Mix**	**Rest**	**Proof**
K1 (+)^a)^	*K. humilis*	2.440.26^bc^	2.380.10^abc^	2.480.10^abc^	2.230.10^cde^	2.400.17^bcd^
K1 (−)^a)^	*L. sanfranciscensis*			2.190.58^c^	1.980.16^e^	2.070.20^d^
K2 (+)	*K. humilis*	2.540.15^bc^	2.210.10^bc^	2.480.11^abc^	2.470.06^abcd^	2.630.10^abc^
K2 (−)	*L. sakei*			2.320.03^bc^	2.140.10^de^	2.220.12^cd^
S1 (+)	*S. cerevisiae*	2.860.26^ab^	2.690.15^ab^	2.850.10^a^	2.680.12^ab^	2.740.09^ab^
S1 (−)	*L. sanfranciscensis*			2.640.20^abc^	2.510.13^abcd^	2.390.11^bcd^
S2 (+)	*S. cerevisiae*	3.120.13^a^	2.820.34^a^	2.730.22^ab^	2.800.22^a^	2.850.16^a^
S2 (−)	*L. sakei*			2.640.14^abc^	2.560.16^abc^	2.570.15^abc^
W1 (+)	*W. anomalus*	2.550.16^bc^	2.320.14^abc^	2.510.15^abc^	2.350.11^bcde^	2.490.06^abc^
W1 (−)	*L. sanfranciscensis*			2.320.20^bc^	2.310.11^bcde^	2.240.06^cd^
W2 (+)	*W. anomalus*	2.480.15^bc^	2.440.14^abc^	2.570.12^abc^	2.380.08^bcd^	2.610.13^abc^
W2 (−)	*L. sakei*			2.420.14^abc^	2.110.13^de^	2.290.14^cd^
L. sf (+)	*L. sanfranciscensis*	2.540.12^bc^	2.450.09^abc^	2.440.14^abc^	2.330.09^bcde^	2.430.07^bcd^
L. sk (+)	*L. sakei*	2.480.03^bc^	2.420.04^abc^	2.400.10^abc^	2.360.04^bcde^	2.390.04^bcd^
CA (+)	CA control^b)^	2.030.08^c^	1.980.12^c^	2.320.12^bc^	2.260.05^cde^	2.250.13^cd^
SD (+)	Straight dough	n.a.	n.a.	2.350.13^bc^	2.260.08^cde^	2.530.12^abc^

*Data are presented as means ± standard deviation of triplicate independent fermentation and baking trials. Values in the same column differ significantly if they do not share a common superscript (*P* < 0.05) (*n* = 3). ^*a*^ (+) indicates addition of baker’s yeast to the bread dough; (−) no addition of baker’s yeast; ^*b*^ CA, chemically acidified; and n.a., not available.*

The content of total peroxides in sourdoughs and bread doughs (Table 4) was inversely related to the thiol content and thus supports the results of thiol analysis. The peroxide levels were highest in chemically acidified doughs when compared to sourdoughs fermented with lactobacilli alone or in combination with other yeasts. Low peroxide levels were observed particularly in sourdoughs fermented with *S. cerevisiae* and *L. sanfranciscensis*. In bread doughs, peroxide levels were lowest in the straight dough control fermented only with baker’s yeast, suggesting that bacterial metabolism contributes to peroxide formation, or attenuates peroxide reduction by yeasts.

**TABLE 4:** Concentration of total peroxides in sourdoughs and bread dough.

**Bread code**	**Combined starters**	**Total peroxides content (μmol/g)**				
		**Stage I**	**Stage II**	**Mix**	**Rest**	**Proof**
K1 (+)^a)^	*K. humilis*	19.430.27^cde^	12.780.25^cd^	10.630.31^d^	11.940.47^cdef^	11.190.52^bcd^
K1 (−)^a)^	*L. sanfranciscensis*			11.160.57^cd^	12.030.46^cdef^	11.340.90^bcd^
K2 (+)	*K. humilis*	19.970.51^cd^	13.970.44^bc^	12.210.95^bcd^	12.060.31^cde^	11.550.65^bcd^
K2 (−)	*L. sakei*			13.100.74^abc^	11.640.21^def^	10.830.32^bcd^
S1 (+)	*S. cerevisiae*	18.540.38^e^	11.220.31^d^	11.160.41^cd^	11.130.53^ef^	9.700.34^de^
S1 (−)	*L. sanfranciscensis*			10.840.92^d^	10.630.53^f^	8.380.88^e^
S2 (+)	*S. cerevisiae*	20.600.32^c^	13.250.20^c^	12.570.26^bcd^	12.600.30^bcd^	12.870.50^ab^
S2 (−)	*L. sakei*			12.330.33^bcd^	13.220.43^bc^	13.580.13^a^
W1 (+)	*W. anomalus*	19.040.53^de^	12.360.47^cd^	11.700.50^bcd^	11.430.28^def^	11.160.23^bcd^
W1 (−)	*L. sanfranciscensis*			12.540.36^bcd^	12.300.29^bcde^	11.940.36^abc^
W2 (+)	*W. anomalus*	19.340.45^cde^	12.540.17^cd^	11.610.68^bcd^	11.910.67^cdef^	10.060.54^cde^
W2 (−)	*L. sakei*			12.570.61^bcd^	12.450.52^bcde^	10.690.80^cd^
L. sf (+)	*L. sanfranciscensis*	22.630.56^b^	14.240.48^b^	14.841.40^a^	14.600.50^a^	13.760.53^a^
L. sk (+)	*L. sakei*	20.030.28^cd^	13.130.35^c^	13.160.52^abc^	13.610.39^ab^	11.671.29^bcd^
CA	CA control^b)^	28.780.33^a^	32.331.34^a^	13.670.11^ab^	13.190.43^bc^	10.840.29^bcd^
SD	Straight dough	n.a.	n.a.	7.850.41^e^	7.100.37^g^	5.220.38^f^

*Data are presented as means ± standard deviation of triplicate independent fermentation and baking trials. Values in the same column differ significantly if they do not share a common superscript (*P* < 0.05) (*n* = 3). ^*a*^ (+) indicates addition of baker’s yeast to the bread dough; (−) no addition of baker’s yeast ^*b*^ CA, chemically acidified; and n.a., not available.*

### Influence of Dough Microbiota on Gluten Polymerization

**FIGURE 2:**
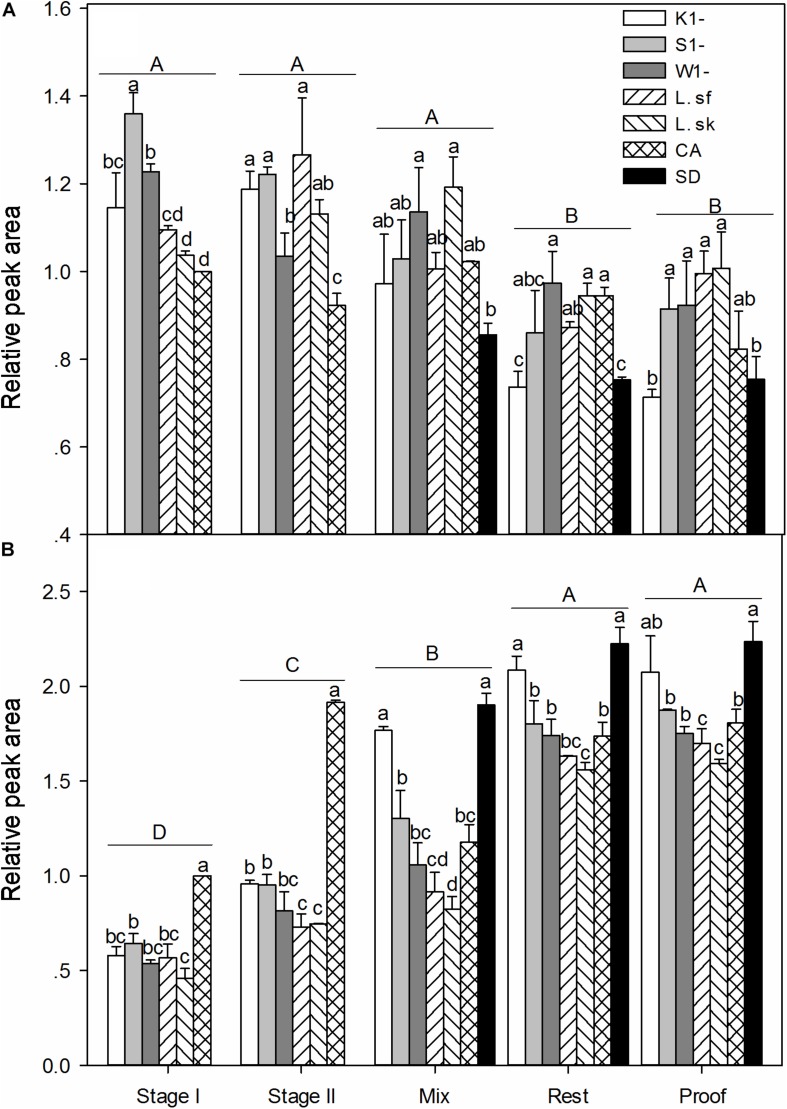
Relative amounts of SDS-soluble proteins (≥91 kDa) **(A)** and SDS-DTT soluble proteins (≥91 kDa) **(B)** in sourdoughs and bread doughs. The results are presented as means ± standard deviation of two independent fermentations and baking trials. Significant differences between fermentation stages are indicated by capital letters; data for different doughs obtained at the same fermentation stage differ significantly (*P* < 0.05) if bars do not share a common lowercase letter.

The content of polymeric SDS-soluble and SDS-DTT soluble proteins was expressed relative to the peak area in the chemically acidified doughs of the same batch (Xu et al., 2018). In sourdoughs fermented with *K. humilis*, *S. cerevisiae*, and *W. anomalus*, the relative content of SDS or SDS-DTT soluble polymeric gluten proteins was not different after fermentation of these yeasts with *L. sanfranciscensis* or *L. sakei* (Supplementary Tables S1, S2). In bread doughs, fermentation of a specific sourdough with or without addition of baker’s yeast also did not impact gluten polymerization. Figure 2 thus depicts only the gluten content of sourdoughs fermented with yeast and *L. sanfranciscensis* and of bread doughs fermented without baker’s yeast if yeasts were added to the sourdough; the complete data set is shown in Supplementary Tables S1, S2. The content of SDS-DTT soluble polymeric gluten proteins increased during processing and was highest after the dough rest and proofing; a corresponding decrease in the relative content of SDS-soluble polymeric gluten proteins was observed during processing (Figures 2A,B). In sourdoughs, the relative content of SDS-DTT soluble gluten proteins was highest in the chemically acidified control; sourdoughs generally exhibited a lower content of SDS-DTT soluble gluten proteins and a higher content of SDS-soluble polymeric gluten proteins. In bread doughs, the straight dough control and doughs fermented with *K. humilis* exhibited the highest content of SDS-DTT soluble proteins and correspondingly a lower of SDS-soluble polymeric gluten proteins.

### Influence of Dough Microbiota on Volatile Compounds of Bread Crumb

**FIGURE 3:**
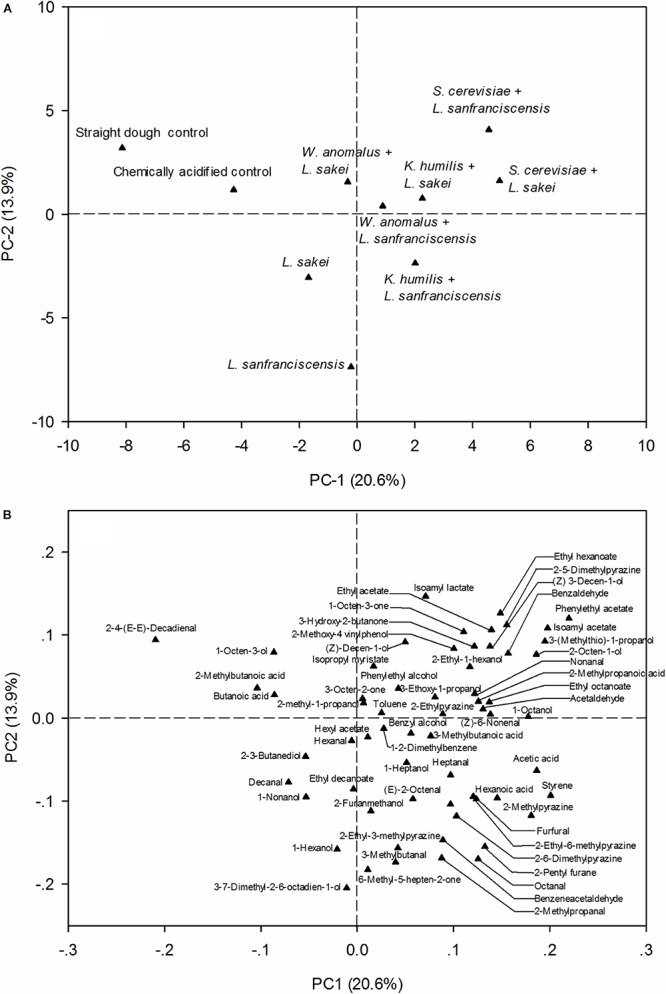
The score plot **(A)** and loading plot **(B)** showing the influence of different starter cultures on the volatile compounds of bread crumb.

In this study, a total of 62 volatile compounds were identified in bread crumb; these compounds include some of the major flavor volatiles of wheat bread crumb (Hansen and Schieberle, 2005) as well as compounds that are generated or consumed in the same metabolic or chemical pathway as flavor compounds. The initial analysis of the volatile pattern was based on principal component analysis (Figure 3A). Breads fermented with different fermentation microbiota were well separated. Breads produced without lactobacilli clustered in the upper left quadrant of the plot; breads produced without sourdough yeasts clustered in the lower left quadrant of the plot while breads produced with sourdough yeasts and lactobacilli clustered in the right quadrants. The loading plot indicates that the chemically acidified bread contained higher quantities of (E,E)-2,4-decadienal, butanoic acid (sweaty, rancid) and 2-methylbutanoic acid (sweaty, cheesy) than other breads (Figure 3B). *L. sakei* breads were associated with 2,3-butanediol while *L. sanfranciscensis* breads associated with 1-nonanol and 1-hexanol, alcohols that are produced by reduction of aldehydes resulting from lipid oxidation. 2-Octen-1-ol (green), benzyl alcohol (pleasant aromatic), and 2-ethyl-1-hexanol (green, vegetable) were detected only from bread made with mixed starters. Pyrazines, Maillard reaction products with “roasted” and “popcorn-like” flavor that are produced in the crust during baking, were also associated with sourdoughs fermented by both yeasts and lactobacilli.

To analyze differences between the three yeasts in major metabolic pathways related to flavor formation, bread crumb volatiles related to carbohydrate metabolism, amino acid metabolism, lipid oxidation and ester formation are shown in Table 5. Data obtained with the same yeast but with different lactobacilli was averaged to highlight differences between yeast species; moreover, data obtained with chemically acidified dough, straight dough, and sourdough fermented with lactobacilli and baker’s yeast are shown for comparison. Generally, volatile levels were lowest in bread produced with straight dough process and highest in sourdoughs fermented with yeasts and lactobacilli. Acetaldehyde was detected only in breads with sourdough additions; levels were highest in sourdoughs fermented with *S. cerevisiae*. All samples contained volatiles that are produced from amino acids by yeast metabolism; the relative concentrations were lowest in straight dough bread and highest when *S. cerevisiae* was used for sourdough fermentation. Volatiles related to lipid oxidation were higher in sourdough bread than in straight dough bread but differences between yeasts were observed only for few volatiles. The presence of esters in the bread crumb was increased by sourdough fermentation and strongly dependent on the yeast strain used for fermentation. *S. cerevisiae* fermented sourdough bread contained high levels of ethyl acetate, ethyl hexanoate, ethyl octanoate, isoamyl acetate, isoamyl lactate, and phenylethyl acetate; *K. humilis* produced high levels of isopropyl myristate. *W. anomalus* produced hexyl acetate and ethyl decanoate.

**TABLE 5:** Volatile compounds of bread crumb.

**Compounds**	**Chemically acidified**	**Straight dough**	**LAB**	***K. humilis***	***S. cerevisiae***	***W. anomalus***
**Volatiles related to carbohydrate metabolism**						
Acetaldehyde	10110^b^	–	146 ± 52^b^	17532^b^	73854^a^	14243^b^
Acetic acid	3202327^a^	–	3906 ± 1193^a^	56451729^a^	51501373^a^	52322369^a^
Acetoine	26962^a^	22822^a^	166 ± 54^a^	491248^a^	410101^a^	28678^a^
**Volatiles related to amino acid metabolism**						
2-Methyl-1-propanol	1496213^ab^	1570150^a^	744 ± 148^c^	1245255^ab^	1506151^a^	1096219^b^
2-Methylpropanal	68988^a^	–	812 ± 84^a^	600150^a^	505210^a^	574189^a^
2-Methylpropanoic acid	17530^ab^	72^c^	97 ± 45^b^	19954^a^	16543^ab^	974^bc^
3-Methyl-1-butanol	137152785^a^	12535235^a^	13719 ± 2408^a^	149752907^a^	149504435^a^	11115916^a^
3-Methylbutanal	4843527^cd^	3087102^d^	10650 ± 769^a^	6640770^bc^	74901300^b^	77701900^b^
3-Methylbutanoic acid	–	–	12 ± 1^c^	24026^b^	32730^a^	–
Phenylethanol	7115694^b^	572892^c^	4254 ± 545^d^	6770369^b^	9868120^a^	5284346^c^
3-(Methylthio)-1-propanol	29019^b^	497^c^	354 ± 64^b^	36035^b^	1031166^a^	34258^b^
**Volatiles relate to lipid oxidation**						
1-Hexanol	2365345^a^	194054^a^	2274 ± 299^a^	1817134^b^	1260152^c^	1425202^c^
Hexanal	3104595^a^	2939469^a^	2804 ± 275^a^	3330600^a^	2320140^a^	2530630^a^
Hexanoic acid	7123^c^	5.32^c^	395 ± 47^ab^	520204^a^	29471^b^	37139^b^
1-Heptanol	–	–	99 ± 1^a^	314196^a^	–	30442^a^
Heptanal	50316^a^	48336^a^	605 ± 47^a^	63636^a^	54340^a^	633100^a^
2-(E)-Heptenal	57369^a^	27645^c^	605 ± 20^a^	42029^b^	57140^a^	56240^a^
1-Octanol	1424^bc^	908^d^	114 ± 24^cd^	18813^a^	16012^b^	12721^c^
Octanal	3014^bc^	1447^c^	477 ± 37^a^	37565^ab^	402139^ab^	24915^c^
Octanoic acid	46	–	–	9930	–	–
(E)-2-Octenal	3251^a^	1647^c^	335 ± 31^a^	27154^ab^	31136^a^	23854^bc^
1-Nonanol	–	34325^a^	262 ± 55^a^	258198^a^	16958^a^	20536^a^
Nonanal	97258^a^	87234^a^	913 ± 59^a^	1120110^a^	116076^a^	93834^a^
(Z)-6-Nonenal	29980^b^	1399^c^	361 ± 59^ab^	44355^a^	42050^a^	39052^ab^
Decanal	24740^a^	14013^d^	217 ± 15^ab^	16215^cd^	14912^d^	18623^bc^
2,4-(E,E)-Decadienal	182^a^	223^a^	–	–	–	–
**Esters**						
Ethyl acetate	18310^b^	1125^b^	187 ± 7^b^	13127^b^	822110^a^	813160^a^
Isoamyl acetate	23819^c^	5211^c^	233 ± 27^c^	580140^b^	1230230^a^	58382^b^
Isoamyl lactate	–	–	–	29852^b^	52633^a^	11623^c^
Ethyl hexanoate	8611^b^	423^b^	103 ± 30^b^	8321^b^	413130^a^	31755^a^
Hexyl acetate	–	–	33 ± 8^b^	–	–	92.221^a^
Ethyl octanoate	3967^b^	37124^b^	734 ± 251^ab^	529100^b^	939280^a^	586138^b^
Ethyl decanoate	336^c^	3.71^d^	68 ± 4^a^	50.69.0^b^	–	76.88.2^a^
Phenylethyl acetate	19312^d^	1263^d^	208 ± 39^d^	51562^b^	77382^a^	38014^c^
Isopropyl myristate	7331^c^	443^c^	66 ± 34^c^	52833^a^	82.443^c^	25732^b^

*Shown is the relative peak area × 10^6^. Data are presented as means ± standard deviation of 4 independent fermentation and baking trials. Values in the same row differ significantly if they do not share a common superscript (*P* < 0.05). Chemically acidified: chemically acidified sourdough bread control. Straight dough: straight dough bread control. LAB: sourdough bread fermented with *L. sanfranciscensis* and *L. sakei*. *K. humilis*: sourdough bread fermented with *K. humilis* + *L. sanfranciscensis* and *K. humilis* + *L. sakei*. *S. cerevisiae*: sourdough bread fermented with *S. cerevisiae* + *L. sanfranciscensis* and *S. cerevisiae* + *L. sakei. W. anomalus*: sourdough bread fermented with *W. anomalus* + *L. sanfranciscensis* and *W. anomalus* + *L. sakei.* –: Not detected.*

## Discussion

In traditional sourdoughs used as sole leavening agents, both yeasts and heterofermentative lactobacilli contribute to CO_2_ production and leavening (Brandt et al., 2004). Some authors suggested that yeasts and heterofermentative lactobacilli equally contribute to CO_2_ production while others attributed CO_2_ production mainly to yeast activity (Gobbetti et al., 1995; Brandt et al., 2004). Both scenarios likely match conditions in artisanal practice where, depending on the fermentation conditions, the ratio of cell counts of yeasts to lactobacilli ranges from 1:10 to 1:100 (Gänzle et al., 1998). Because yeasts have a much higher surface area and metabolic activity per cell, CO_2_ production is mainly attributable to yeast activity if the ratio of cell counts is at one extreme of the spectrum while lactobacilli significantly contribute when cell counts of yeasts are lower (Brandt et al., 2004). Industrial bakeries generally use sourdough in conjunction with addition of baker’s yeast to minimize the risk of fermentation failure. Remarkably, the use of sourdough enhanced the leavening capacity of baker’s yeast but this effect was observed only in sourdoughs containing yeasts. With few exceptions, the CO_2_ production of yeasts was highly correlated to an increased bread volume and a reduction of bread hardness.

Only few studies compared the leavening capacity of different sourdough yeasts (Gobbetti et al., 1995; Häggman and Salovaara, 2008; Reale et al., 2013); and past studies did not relate CO_2_ production to the impact of yeast on gluten cross-linking. Differences between *W. anomalus*, *K. humilis*, and *S. cerevisiae* may not be representative for the respective species in general because CO_2_ production by different strains in sourdough is strain specific and dependent on the fermentation conditions (Häggman and Salovaara, 2008). More importantly, *S. cerevisiae* had a strong influence on the concentration of free thiols in sourdough and bread dough, which were highest in doughs fermented with *S. cerevisiae* (Tang et al., 2017), and on the concentration of peroxides, which were lowest in doughs fermented with *S. cerevisiae*. Earlier studies indicated that the disulfide-mediated crosslinking of gluten proteins is a dynamic process (Weegels et al., 1996). Gluten depolymerization through disulfide-exchange reactions during dough rest and proofing may result in an increased bread volume in specific cases (Xu et al., 2018). Other yeast metabolites may also affect the viscoelastic properties of wheat dough but experimental documentation for their contribution to bread volume is weak (Rezaei et al., 2015). The use of sourdoughs increased bread volume and decreased crumb hardness relative to a chemically acidified dough. CO_2_ production by baker’s yeast was identical in these doughs but peroxide and thiol levels were highest and lowest, respectively, in chemically acidified doughs; this further corroborates a contribution of low-molecular weight thiol compounds to improved bread volume in long fermentation processes (Xu et al., 2018). The relative abundance of polymeric gluten proteins in dough showed no clear relationships to bread volume and crumb hardness, likely because of overlapping effects of proteolysis, reductive depolymerization, and CO_2_ formation (Gänzle, 2014).

Solid-phase microextraction and gas chromatography/mass spectrometry analysis as used in this study does not provide absolute quantification of volatiles but are a suitable technique to compare volatiles produced by different microorganisms (Demyttenaere et al., 2004; Pétel et al., 2016; Di Renzo et al., 2018). The flavor of bread crumb is determined mainly by enzymatic and microbial conversions at the dough stage; Maillard reaction products are much less relevant when compared to the bread crust (Hansen and Schieberle, 2005; Gänzle, 2014; Pétel et al., 2016). Overall, the spectrum and the relative abundance of (flavor) volatiles was highest when sourdoughs were fermented with yeasts and lactobacilli, confirming the general observation that diverse fermentation microbiota result in diverse and intense food flavor (Gänzle, 2019). The relative abundance of aldehydes or alcohols originating from lipid oxidation was more strongly influenced by lactic metabolism than by yeast activity, confirming to the ability of heterofermentative lactobacilli to reduce flavor-active aldehydes to the corresponding alcohols with a lower odor intensity (Vermeulen et al., 2007). The modest impact of *S. cerevisiae* on peroxide levels in dough had no apparent repercussions on volatile products of lipid peroxidation (Tables 4, 5). Flavor formation through carbohydrate metabolism indicated a strong interdependence of yeast and lactic metabolism. Acetate formation was highest when sourdoughs were fermented with yeasts and lactobacilli; this observation reflects the hydrolysis of wheat fructans by yeast invertase, followed by fructose conversion to mannitol with concomitant acetate formation by heterofermentative lactobacilli (Gänzle, 2014). Reasons for the high level of acetaldehyde in *S. cerevisiae* and LAB fermented sourdoughs remain unexplored. In yeasts, the main metabolic pathways for flavor formation are the conversion of pyruvate to diacetyl, the conversion of amino acids to flavor volatiles via the Ehrlich pathway, conversion of ferulic acid to vinyl- or ethylguaiacol, and formation of esters (Dzialo et al., 2017). Flavor formation via the Ehrlich pathway was strongly enhanced by sourdough fermentation because proteolysis in sourdough releases the precursor amino amino acids through proteolysis (Gassenmeier and Schieberle, 1995; Thiele et al., 2002). Among the yeasts, *S. cerevisiae* had the highest capacity for transamination of methionine and phenylalanine (this study); these amino acids are converted to 3-methylthiopropanol and phenylethanol, respectively, in the Ehrlich pathway (Hansen and Schieberle, 2005).

Metabolic differences between yeasts were highest with respect to the formation of esters. In *S. cerevisiae*, Aft1/Atf2 synthesize acetate esters of higher alcohols while Eeb1/Eht1 catalyze ester formation with ethanol and long-chain fatty acids as substrates (Dzialo et al., 2017). The synthesis of aromatic esters attracts insects as vectors to achieve dispersal of the population (Christiaens et al., 2014). Ester synthesis by yeasts is species- or strain specific, likely reflecting preferences for different insect vectors (Christiaens et al., 2014; Dzialo et al., 2017). The relative abundance of isoamyl acetate (banana-like), isoamyl lactate (fruity, nutty), and phenylethyl acetate (rose) was highest in bread made from sourdough fermented with *S. cerevisiae*. Isopropyl myristate and hexyl acetate (green, fruity) were identified only in bread produced with *K. humilis* and *W. anomalus*. Strain-specific ester formation by brewer’s yeasts contributes to the distinct flavor of individual products but ester formation by yeasts is not widely employed for improved bread quality.

## Conclusion

In conclusion, the present study confirms that the role of thiol exchange reactions on bread quality differs between long fermentation protocols including lactobacilli and short fermentations with baker’s yeast only. The work further documents the role of different sourdough yeasts on thiol exchange reactions in wheat dough, the volume of the resulting bread, and the conversion of dough components to volatiles. In the past century, recipes and processes for industrial bread production were optimized mainly for short processes with straight dough fermentation. Currently, the baking industry reverts back to long fermentation processes to take advantage of the beneficial impact of sourdough lactobacilli and yeasts on bread quality. The present study provides an important basis for optimization of processes and recipes for slow fermentations.

## Data Availability

The raw data supporting the conclusions of this manuscript will be made available by the authors, without undue reservation, to any qualified researcher.

## Author Contributions

DX carried out the experiments and wrote the manuscript. YZ performed biochemical analyses on the sourdoughs. KT performed statistical analyses and revised the manuscript. YH directed the experimental phases and revised the manuscript. XX made funds available for the research costs. MG ideated the study and revised the manuscript.

## Conflict of Interest Statement

The authors declare that the research was conducted in the absence of any commercial or financial relationships that could be construed as a potential conflict of interest.
