# Supplementary material for: Effect of Mixed Cultures of Yeast and Lactobacilli on the Quality of Wheat Sourdough Bread
Source: Front Microbiol. 2019 Sep 10;10:2113. doi: 10.3389/fmicb.2019.02113 (PMC6746982; doi:10.3389/fmicb.2019.02113)
Supplement: Supplementary file 1 [file Table_1.DOCX]

Supplementary Material

## Supplementary Tables

**Supplementary Table S1.** Relative amounts of SDS-soluble proteins (≥ 91 kDa) in sourdoughs and bread dough. Data are presented as means ± standard deviation of two independent fermentation and baking trials. Values in the same column differ significantly if they do not share a common lower case superscript (*P*<0.05) (n=2).

| Addition of Baker’s yeast | Combined starters | Relative content of SDS-soluble proteins | | | | |
| --- | --- | --- | --- | --- | --- | --- |
|  |  | Stage I | Stage II | Mix | Rest | Proof |
| K1 (+)^a)^ | *K. humilis L. sanfranciscensis* | 1.15±0.08^cde^ | 1.19±0.04^a^ | 0.97±0.15^b^ | 0.73±0.05^c^ | 0.76±0.05^cd^ |
| K1 (-) |  |  |  | 0.97±0.11^b^ | 0.74±0.04^c^ | 0.71±0.02^d^ |
| K2 (+) | *K. humilis  L. sakei* | 1.14±0.02^de^ | 1.17±0.14^a^ | 1.03±0.06^ab^ | 0.84±0.05^b^ | 0.91±0.06^ab^ |
| K2 (-) |  |  |  | 0.95±0.03^b^ | 0.83±0.09^b^ | 0.80±0.02^c^ |
| S1 (+) | *S. cerevisiae  L. sanfranciscensis* | 1.36±0.05^a^ | 1.22±0.02^a^ | 1.03±0.16^ab^ | 0.92±0.10^ab^ | 0.88±0.02^b^ |
| S1 (-) |  |  |  | 1.03±0.08^ab^ | 0.86±0.09^b^ | 0.92±0.07^ab^ |
| S2 (+) | *S. cerevisiae  L. sakei* | 1.31±0.03^ab^ | 1.18±0.01^a^ | 0.98±0.11^b^ | 1.00±0.08^a^ | 0.90±0.03^ab^ |
| S2 (-) |  |  |  | 1.04±0.10^ab^ | 0.89±0.10^ab^ | 0.86±0.09^abc^ |
| W1 (+) | *W. anomalus  L. sanfranciscensis* | 1.23±0.02^bcd^ | 1.04±0.05^ab^ | 1.02±0.02^b^ | 0.95±0.07^ab^ | 0.84±0.09^b^ |
| W1 (-) |  |  |  | 1.14±0.10^ab^ | 0.97±0.07^ab^ | 0.92±0.10^ab^ |
| W2 (+) | *W. anomalus  L. sakei* | 1.27±0.05^abc^ | 1.14±0.03^ab^ | 1.02±0.04^b^ | 0.90±0.01^b^ | 0.90±0.01^a^ |
| W2 (-) |  |  |  | 1.10±0.06^ab^ | 0.93±0.02^ab^ | 0.89±0.09^ab^ |
| L. sf (+) | *L. sanfranciscensis* | 1.10±0.01^ef^ | 1.27±0.13^a^ | 1.01±0.04^b^ | 0.87±0.01^b^ | 1.00±0.05^a^ |
| L. sk(+) | *L. sakei* | 1.04±0.01^ef^ | 1.13±0.03^ab^ | 1.19±0.07^a^ | 0.95±0.03^ab^ | 1.01±0.08^a^ |
| CA | CA control^b)^ | 1.00±0.00^f^ | 0.92±0.03^b^ | 1.02±0.01^b^ | 0.95±0.02^ab^ | 0.82±0.09^bc^ |
| SD | Straight dough | n.a. | n.a. | 0.86±0.03^c^ | 0.75±0.01^c^ | 0.75±0.05^cd^ |

^a)^ (+) indicates addition of baker’s yeast to the bread dough; (-) no addition of baker’s yeast

^b)^ CA, chemically acidified

**Supplementary Table S2.** Relative amounts of SDS-DTT soluble proteins (≥ 91 kDa) in sourdoughs and bread dough. Data are presented as means ± standard deviation of two independent fermentation and baking trials. Values in the same column differ significantly if they do not share a common lower case superscript (*P*<0.05) (n=2)

| Addition of Baker’s yeast | Combined starters | Relative content of SDS-DTT soluble proteins | | | | |  |
| --- | --- | --- | --- | --- | --- | --- | --- |
|  |  | Stage I | Stage II | Mix | Rest | Proof | |
| K1 (+)^a)^ | *K. humilis L. sanfranciscensis* | 0.58±0.05^bc^ | 0.96±0.02^bc^ | 1.66±0.01^b^ | 2.00±0.10^bc^ | 1.82±0.11^bc^ | |
| K1 (-) |  |  |  | 1.77±0.02^ab^ | 2.08±0.07^ab^ | 2.07±0.19^ab^ | |
| K2 (+) | *K. humilis  L. sakei* | 0.52±0.06^bc^ | 0.78±0.05^d^ | 1.27±0.15^c^ | 1.94±0.09^bcd^ | 1.83±0.22^bcd^ | |
| K2 (-) |  |  |  | 1.27±0.09^c^ | 1.99±0.06^bc^ | 2.05±0.25^ab^ | |
| S1 (+) | *S. cerevisiae  L. sanfranciscensis* | 0.64±0.05^b^ | 0.95±0.05^bc^ | 0.76±0.03^g^ | 1.60±0.03^f^ | 1.75±0.08^cd^ | |
| S1 (-) |  |  |  | 1.30±0.15^c^ | 1.80±0.12^cde^ | 1.87±0.01^b^ | |
| S2 (+) | *S. cerevisiae  L. sakei* | 0.67±0.12^b^ | 1.02±0.01^b^ | 1.08±0.01^cde^ | 1.77±0.06^def^ | 1.76±0.03^cd^ | |
| S2 (-) |  |  |  | 1.19±0.03^c^ | 1.89±0.06^bcd^ | 2.10±0.08^ab^ | |
| W1 (+) | *W. anomalus  L. sanfranciscensis* | 0.54±0.02^bc^ | 0.81±0.10^cd^ | 1.13±0.04^cd^ | 1.70±0.16^ef^ | 1.88±0.16^bcd^ | |
| W1 (-) |  |  |  | 1.06±0.12^cdef^ | 1.74±0.09^def^ | 1.75±0.04^c^ | |
| W2 (+) | *W. anomalus  L. sakei* | 0.53±0.01^c^ | 0.70±0.02^d^ | 0.88±0.03^efg^ | 1.58±0.05^f^ | 1.72±0.01^cd^ | |
| W2 (-) |  |  |  | 0.81±0.02^g^ | 1.85±0.11^cde^ | 1.92±0.09^bc^ | |
| L. sf (+) | *L. sanfranciscensis* | 0.57±0.07^b^ | 0.73±0.07^d^ | 0.92±0.10^defg^ | 1.63±0.01^ef^ | 1.70±0.10^de^ | |
| L. sk(+) | *L. sakei* | 0.46±0.05^d^ | 0.74±0.01^d^ | 0.82±0.07^g^ | 1.56±0.04^f^ | 1.59±0.02^e^ | |
| CA | CA control^b)^ | 1.00±0.00^a^ | 1.92±0.01^a^ | 1.18±0.09^c^ | 1.74±0.07^de^ | 1.81±0.07^bcd^ | |
| SD | Straight dough | n.a. | n.a. | 1.90±0.06^a^ | 2.22±0.09^a^ | 2.24±0.10^a^ | |

^a)^ (+) indicates addition of baker’s yeast to the bread dough; (-) no addition of baker’s yeast

^b)^ CA, chemically acidified
